# Physical activity may compensate for prolonged TV time regarding pulse rate—a cross-sectional study

**DOI:** 10.1080/03009734.2018.1540505

**Published:** 2018-11-23

**Authors:** Kristina Beijer, Erik Lampa, Johan Sundström, Peter M. Nilsson, Sölve Elmståhl, Nancy L. Pedersen, Lars Lind

**Affiliations:** a Department of Medical Sciences, Uppsala University, Uppsala University Hospital, UppsalaSweden;; b Uppsala Clinical Research Center (UCR), Uppsala, Sweden;; c Department of Clinical Sciences, SUS Malmö, Malmö, Sweden;; d Division of Geriatric Medicine, Department of Health Sciences, Lund University, Malmö University Hospital, Malmö, Sweden;; e Department of Medical Epidemiology and Biostatistics, Karolinska Institute, Stockholm, Sweden

**Keywords:** Cross-sectional study, epidemiology, exercise, pulse rate, sitting time, television

## Abstract

*Background*. Regular exercise reduces pulse rate, but it is less clear how prolonged sitting time affects pulse rate. Our hypothesis was that high physical activity could compensate for prolonged sitting time regarding the pulse rate.

*Methods*. Regression analysis was performed on cross-sectional data including 47,457 men and women based on two Swedish cohort studies, EpiHealth (18–45 years) and LifeGene (45–75 years). Self-reported leisure time physical activity was given in five levels, from low (level 1) to vigorous (level 5), and television time was used as a proxy of sitting time.

*Results*. A higher physical activity (level 4 compared to level 1) was associated with a lower pulse rate in middle-aged females (-2.7 beats per minute [bpm]; 95% CI -3.3 to -2.2) and males (-4.0 bpm; 95% CI -4.7 to -3.4). The relationship between physical activity and pulse rate was strongest in the young. A prolonged television time (3 h compared to 1 h per day) was associated with a slightly higher pulse rate in middle-aged females (+0.6 bpm; 95% CI +0.3 to +0.8) and males (+0.9 bpm; 95% CI +0.7 to +1.2). Among participants with a prolonged television time (3 h), those with a high physical activity (level 4) had a lower pulse rate compared to those with a low physical activity (level 1).

*Conclusions*. A prolonged television time was associated with a high pulse rate, while high physical activity was associated with a low pulse rate. The results suggest that a high physical activity could compensate for a prolonged television time regarding pulse rate.

## Introduction

A high resting pulse rate is associated with cardiovascular diseases, such as atherosclerosis, coronary artery disease, myocardial infarction, and heart failure, and is a predictor of cardiovascular and all-cause mortality (1–6).

It is well known that engagement in physical activity lowers resting pulse rate (3, 7–12). However, it is not clear how physical inactivity, such as daily sitting time, affects pulse rate, but a prolonged sitting time has been associated with elevated risk of cardiovascular disease, type 2 diabetes mellitus, obesity, and all-cause mortality, relationships being independent of moderate to vigorous physical activity (13–17).

In Western societies, the amount of sitting time is increasing, and therefore it is of great interest to investigate if higher amounts of leisure time physical activity could compensate for a prolonged sitting time. Previous studies have shown that high levels of physical activity attenuate or even eliminate the increased mortality and morbidity risks associated with high total sitting time (18, 19). The objective of the present study was to investigate the interplay between television time (as a proxy for sitting time) and leisure time physical activity regarding resting pulse rate. We further investigated if such interplay between television time and physical activity regarding resting pulse rate was different in males and females, as well as in different age groups. Moreover, we wanted to investigate if physical activity lowers and television time increases resting pulse rate. We also tested the hypothesis that high amounts of leisure time physical activity would counteract a prolonged television time. We used data from two Swedish cohort studies with almost the same protocol in order to obtain data over the main part of the adult age-span (20 to 75 years) in a large number of individuals.

## Methods

Both the EpiHealth (EH, Epidemiology for Health) and LifeGene (LG) cohorts consist of web-based questionnaire assessments, physical measurements, and bio-banking of Swedish residents. The sample used for the analyses consisted of 47,457 individuals (21,847 from the EpiHealth and 25,583 from the LifeGene sample) aged 20–75 years.

### EpiHealth

In short, males and females, aged 45–75 years, were invited to participate in the EpiHealth cohort study; details are found in (20). Participants were randomly selected between 2011 and 2016 from the population registry in the Swedish cities Malmö and Uppsala, and the response rate was 20%.

### LifeGene

Persons aged 18–45 years were randomly recruited between 2009 and 2016 from the general Swedish population, originating mainly from or around Stockholm, with a response rate of approximately 20%. Household members (partner and children) were also encouraged to participate. Participants could also spontaneously register participation on the LifeGene website. For details, see (21).

### Physical examination and pulse rate measurement

Participants visited a test center where pulse rate was recorded by trained staff in the sitting position following 5 min of rest, and the average of two measurements was used. Pulse rate was assessed by an automatic device (Omron M6, Kyoto, Japan) with the left arm and cuff at heart level. A standard 12 cm cuff was used, and other dimensions were available if needed; details can be seen in (20, 21). In EH, atrial fibrillation was determined at the ECG during the physical examination at the EH test center.

### Questionnaire data

In both studies, participants responded to a comprehensive internet-based questionnaire. From the beginning of EH, the idea was to merge EH data with the ongoing LG study. That is why the majority of questions are identical in the two studies. In this study, all questions and variables are identical except for the questions about television time and education level (harmonization is described below).

Physical activity in leisure time was reported in five levels as: 1) mainly sedentary; 2) not defined but positioned between level 1 and level 3; 3) walking 30 min per day; 4) not defined but positioned between level 3 and level 5; and 5) vigorous exercise 60 min per day. All participants, including unemployed and retired persons, reported the occupational physical activity during their daily activities such as work, studies, or other activities. Occupational physical activity was categorized in five levels as: 1) mainly sedentary; 2) not defined, but positioned between level 1 and level 3; 3) active (mainly standing or walking); 4) not defined, but positioned between level 3 and level 5; and 5) very active (hard physical work). The participants were asked for their marital status (coded as married including cohabitation, unmarried), alcohol consumption (never, once a month or less, 2–3 times a month, once a week, 2–3 times a week, 4 times a week or more), tobacco use (current smoker, current non-smoker), and use of antihypertensive medication (yes, no). In LG, participants responded in the questionnaire if they have or have had irregular heartbeat (tachycardia).

Participants estimated the daily time they spent watching TV (television time) on an eight-graded scale in EpiHealth (0–4 min, 5–9 min, 10–19 min, 20–39 min, 40–90 min, 1.5–3 h, 3–6 h, >6 h) and on a six-graded scale in LifeGene (<30 min, 30–59 min, 1–2 h, 3–4 h, 5–8 h, >8 h). In LifeGene, participants also reported how many days per week (1–7 days) they watched TV. In order to harmonize the television time of the two samples, first, television time in hours per day was determined in EH and LG. Secondly, television time in LG was averaged across the week to hours per day. The harmonized television time (hours per day) was used in the analysis.

There were four possible answers to education level in LG: 1) elementary school (nine years); 2) senior high school (10–12 years); 3) university (>12 years); and 4) other. In EH, seven possible answers for education level existed: answers 1) to 4) were identical to LG, followed by: 5) an old form of high school (6 years); 6) another old form of high school (9 years); and 7) do not know/do not want to answer. To conciliate the questions, answers 5) and 6) were added to answer 1) (elementary school), and only answers 1) to 3) were used as categories in the analysis.

### Ethics approval and consent to participate

This study was approved by the regional ethics review board at Uppsala University. All subjects provided informed consent.

### Statistical analyses

A generalized linear regression model (GLM), using a gamma link function was used to assess the associations between television time and physical activity and pulse rate. Sex and age-specific estimates were obtained by allowing television time and physical activity to interact with sex and age, and the models were further adjusted for alcohol consumption, body mass index (BMI), current smoking, education, marital status, occupational physical activity, and use of antihypertensive medication (no information existed on the kind of antihypertensive medication). Participants with atrial fibrillation (*n* = 409) and tachycardia (*n* = 839) were excluded from the analysis in the EH and LG sample, respectively.

Age was modeled using restricted cubic splines with four interior knots, placed at the 5th, 35th, 65th, and 95th percentiles of the age distribution. Television time, physical activity, and alcohol intake were modeled using restricted cubic splines with three interior knots placed at the 10th, 50th, and 90th percentiles of each variable’s distribution. All interaction terms were forced to consist of the linear parts of the spline terms only in order to avoid an overly smooth fit.

The percentage of missing values across the 11 variables included in the analyses varied between 0% and 22%. In total, 17,019 observations (36%) were incomplete. We used multiple imputation (22) to create and analyze 20 multiply imputed datasets. Incomplete variables were imputed under fully conditional specification (23). Model parameters were estimated with the linear models described above to each complete dataset separately. The estimates and their standard errors were then pooled using Rubin’s rules.

The regression model involves high-order interactions, and the results are best understood through visualizations. All figures are adjusted predictions from the model, varying one, two, or three parameters at a time while holding the rest constant at the median or most frequent category. Predicted differences in pulse rate between levels of the independent variables were formed in a similar manner. For the predictions, 10th, 50th, and 90th percentiles of the age distribution were chosen in order to correspond young, middle-aged, and elderly. The model fit for the predictions (pseudo R^2^) was 0.06.

Four sensitivity analyses were performed. The first excluded participants with antihypertensive medication. The second was restricted to participants aged 65 years or less. We studied if the associations differed between employed versus unemployed and retired (>65 years old) participants. Finally, complete-case analyses were also performed for comparison (*n* = 30,438).

All analyses were performed in R version 3.2.4 (24) using the mice package (23).

## Results

### Characteristics of the study participants

In the combined EH and LG sample, the median age of participants, of whom 58% were women, was 47 years (Table I). LG and EH participants had the same pulse rate (median pulse rate was 66 beats per minute [bpm]). EH participants were older and more likely in a relationship. Moreover, they had higher BMI and shorter education than LG participants, and they were more likely to have been prescribed antihypertensive medication.

**Table 1.** Characteristics of study participants in the combined EpiHealth and LifeGene sample (>45,000).

Characteristic	EH (*n* = 21540)	LG (*n* = 25292)	EH and LG (*n* = 46832)
Pulse rate (bpm)	66 (60–73)	66 (60–72)	66 (60–72)
Age (years)	61 (53–67)	34 (27–42)	47 (33–61)
Alcohol consumption (per week)	1 (0.5–2.5)	1 (0.5–2.5)	1 (0.5–2.5)
TV time (h per day)	2.3 (1–2.3)	1.1 (0.5–1.5)	1.3 (0.8–2.3)
BMI (kg/m^2^)	26 (24–29)	24 (22–26)	25 (22–27)
Leisure time physical activity			
Level 1 (mainly sedentary)	4% (841)	4% (759)	4% (1600)
Level 2	22% (4670)	21% (3845)	21% (8515)
Level 3 (30 min walking/day)	41% (8864)	32% (5789)	37% (14653)
Level 4	27% (5822)	33% (6055)	30% (11877)
Level 5 (vigorous activity 1 h/day)	6% (1334)	10% (1754)	8% (3088)
Occupational physical activity			
Level 1 (mainly sedentary)	4% (841)	4% (759)	4% (1600)
Level 2	22% (4670)	21% (3845)	21% (8515)
Level 3 (active)	41% (8864)	32% (5789)	37% (14653)
Level 4	27% (5822)	33% (6055)	30% (11877)
Level 5 (very active)	6% (1334)	10% (1754)	8% (3088)
Education			
≤9 years	18% (3377)	2% (406)	9% (3783)
10–12 years	29% (5533)	22% (4595)	25% (10128)
>12 years	53% (10151)	76% (15928)	65% (26079)
Sex (females)	56% (12325)	60% (15238)	58% (27563)
AH medication	22% (4734)	2% (611)	11% (5345)
Marital status (married)	74% (15903)	63% (14250)	68% (30153)
Smoking	8% (1673)	6% (1466)	7% (3139)

Data are presented as median (interquartile range) or prevalence in percentage (*n*) of pulse rate (bpm), age (years), alcohol consumption (times per week), television time (h per day), BMI (kg/m^2^), self-reported leisure time and occupational physical activity specified in different levels, education level (years), sex, use of AH medication, marital status, and smoking.

AH: antihypertensive; BMI: body mass index; EH: EpiHealth; LG: LifeGene.

### Physical activity

**Figure 1.**
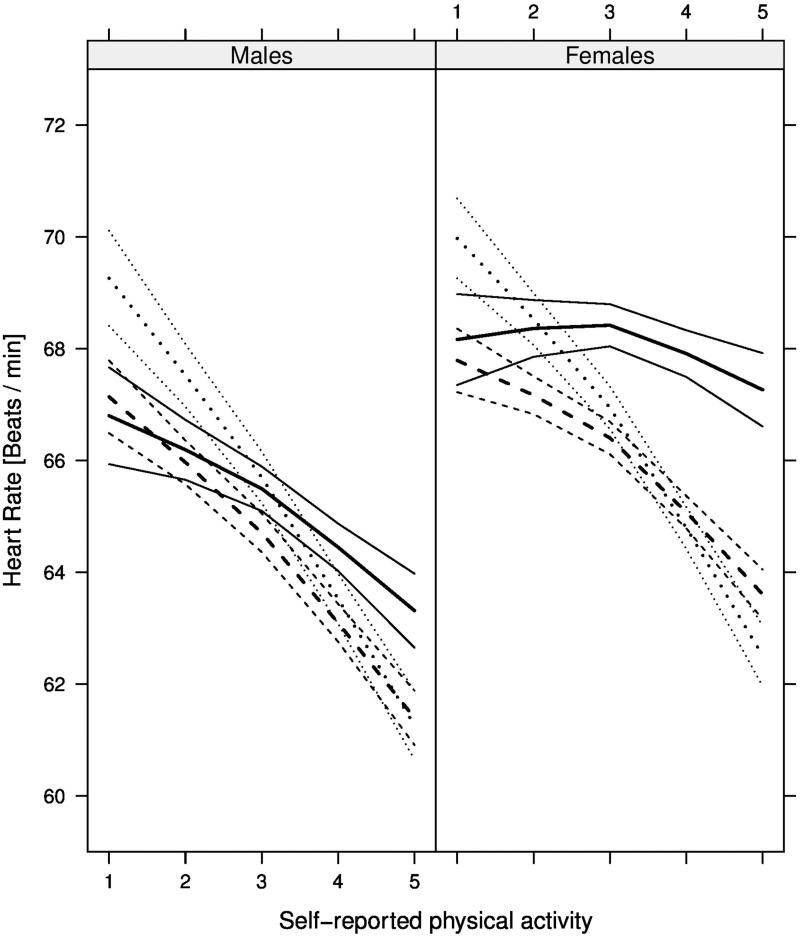
Relationship between resting pulse rate (bpm) and self-reported physical activity (levels) at age 26, 47, and 68 years (*dotted*, *dashed*, and *solid* lines, respectively) with 95% confidence intervals (CI) among 47,457 participants in the combined EpiHealth and LifeGene sample. Estimates are shown for females (right) and males (left) with 1 h television time per day. Physical activity levels are defined as: 1) mainly sedentary; 2) not defined but positioned between level 1 and level 3; 3) walking 30 min per day; 4) not defined but positioned between level 3 and level 5; and 5) vigorous exercise 60 min per day.

Higher physical activity was associated with lower pulse rate in an inverse, non-linear way (*P* < 0.001, Figure 1). For example, a higher physical activity (level 4 compared to level 1) was associated with a lower pulse rate in middle-aged females (-2.7 bpm; 95% CI -3.3 to -2.2) and males (-4.0 bpm; 95% CI -4.7 to -3.4) (47 years and 1 h television time per day) (Table II).

**Table 2.** Pulse rate difference for high versus low physical activity and prolonged versus short television time.

Age, y	Physical activity^a^	TV time	Pulse rate difference, bpm (95% CI)	
			Female	Male
26	Level 4 vs level 1	1 h	−5.2 (-5.9 to -4.5)	−5.7 (-6.6 to -4.9)
47	Level 4 vs level 1	1 h	−2.7 (-3.3 to -2.2)	−4.0 (-4.7 to -3.4)
68	Level 4 vs level 1	1 h	−0.3 (-1.1 to +0.6)	−2.4 (-3.3 to -1.4)
26	Level 3	3 h vs 1 h	+0.6 (+0.2 to +1.0)	+0.4 (-0.1 to +0.9)
47	Level 3	3 h vs 1 h	+0.6 (+0.3 to +0.8)	+0.9 (+0.7 to +1.2)
68	Level 3	3 h vs 1 h	+0.5 (+0.2 to +0.9)	+1.4 (+1.1 to +1.8)

a Physical activity (self-reported): level 1, mostly sitting; level 4, positioned between level 3 and level 5; level 3, walking 30 min per day; level 5, vigorous exercise 60 min per day.

Pulse rate difference between high (level 4) and low (level 1) physical activity in participants with 1 h of television time. Pulse rate difference between prolonged (3 h per day) and short (1 h per day) television time in participants with physical activity level 3. Age-specific predictions (26, 47, and 68 years) are calculated based on 47,457 participants in the combined EpiHealth (18–45 years) and LifeGene (45–75 years) sample.

A significant interaction was observed between physical activity and age regarding resting pulse rate (*P* < 0.001). There was a stronger relationship between physical activity and pulse rate in young compared to old participants (Figure 1, Table II). Visually, this age interaction was particularly obvious in females, although the three-way interaction between physical activity, age, and sex was not statistically significant (*P* = 0.18).

### Television time

**Figure 2.**
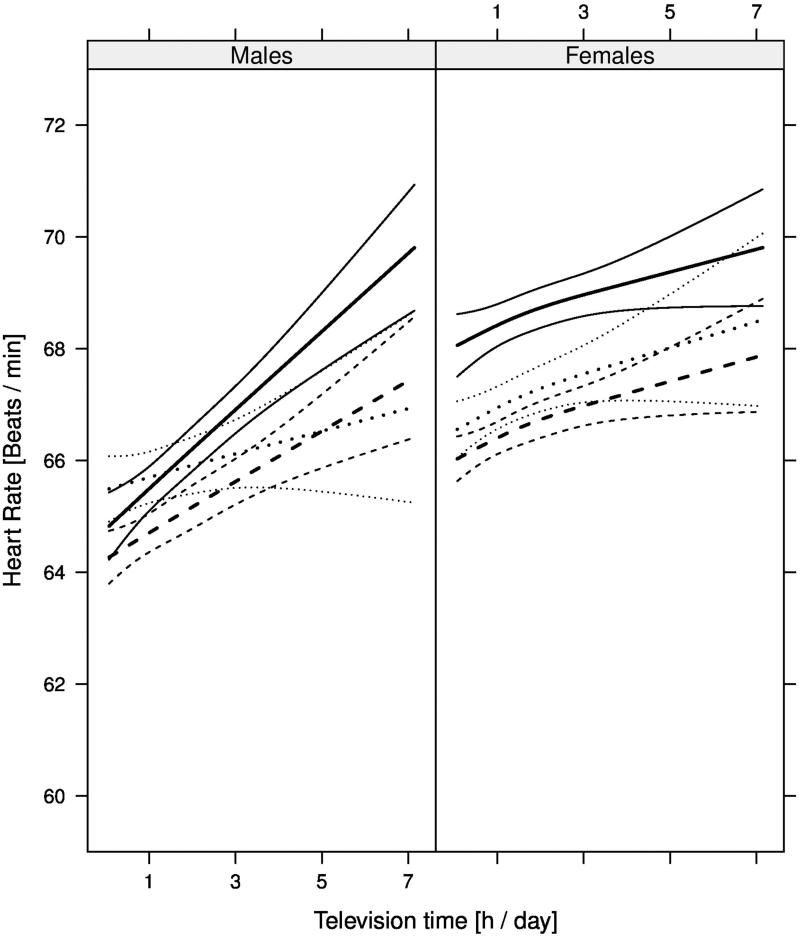
Relationship between resting pulse rate (bpm) and television time (h per day) at age 26, 47, and 68 years (*dotted*, *dashed*, and *solid* lines, respectively) with 95% CI among 47,457 participants in the EpiHealth and LifeGene sample. Estimates are shown for females (right) and males (left) with a physical activity level of 3 (defined as 30 min walking per day).

A longer television time was associated with a higher pulse rate (*P* < 0.001) (Figure 2, Table II), but the relationship was not non-linear (*P* = 0.74). For example, prolonged television time (3 h per day), was associated with a higher pulse rate in middle-aged females (+0.6 bpm; 95% CI +0.3 to +0.8) and males (+0.9 bpm; 95% CI +0.7 to +1.2) (47 years and physical activity level 3).

No interaction (*P* = 0.13) was observed between television time and age regarding pulse rate.

### Physical activity and television time

There was no interaction between physical activity and television time regarding resting pulse rate (*P* = 0.61).

### Combined effects between physical activity and television time

**Figure 3.**
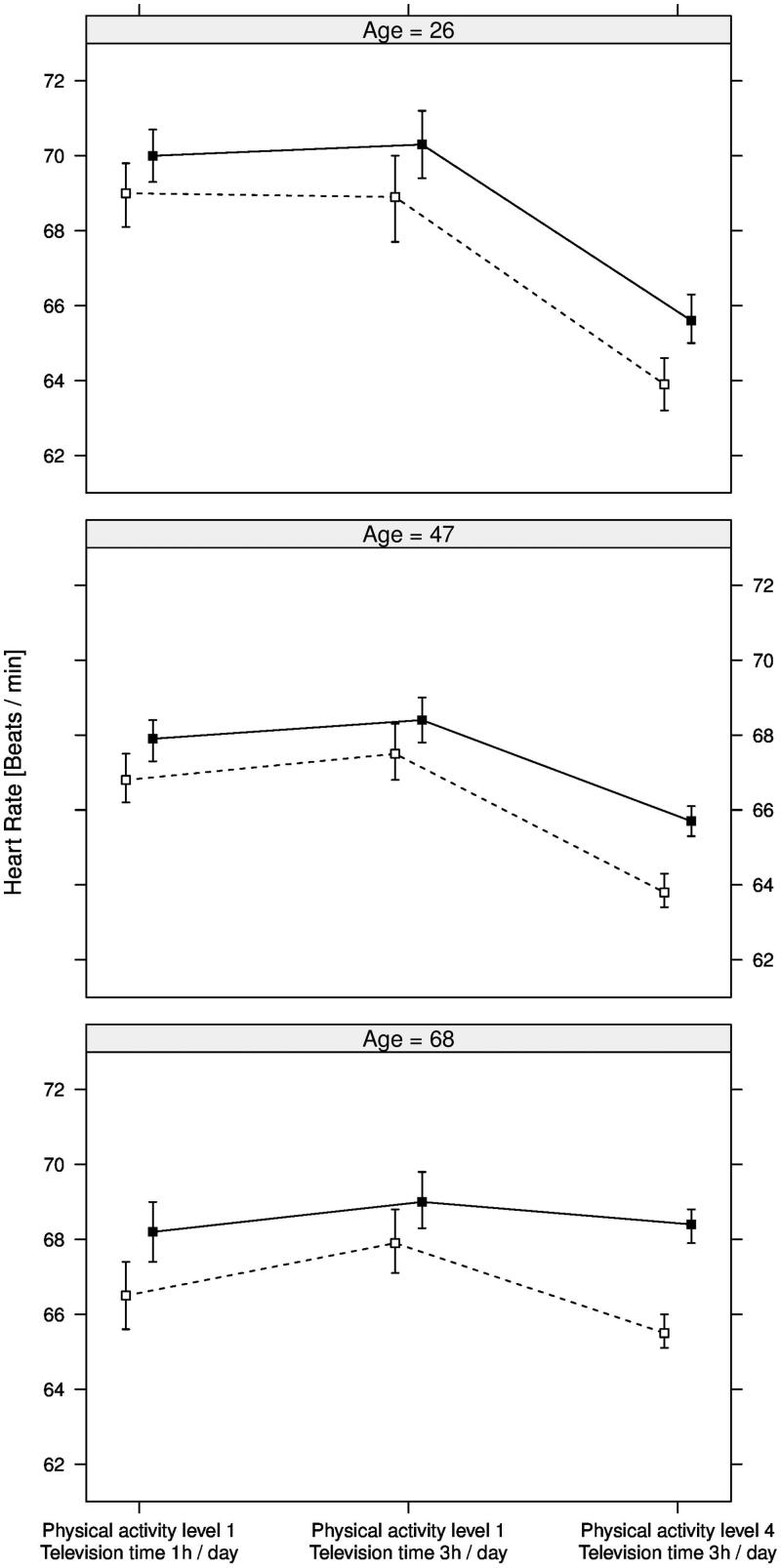
Estimates for resting pulse rate (bpm) with 95% CI (error bars) for age 26, 47, and 68 years and the following three groups of participants (from left to right): Physical activity level 1 and 1 h television time per day; physical activity level 1 and 3 h television time per day; physical activity level 4 and 3 h television time per day. Females are indicated by *filled squares* and males by *unfilled squares*. Physical activity levels are self-reported and defined as: 1) mainly sedentary; 2) not defined but positioned between level 1 and level 3; 3) walking 30 min per day; 4) not defined but positioned between level 3 and level 5; and 5) vigorous exercise 60 min per day. Estimates were calculated based on 47,457 participants in the combined EpiHealth and LifeGene sample.

The combined, and independent, effects between physical activity and television time regarding resting pulse rate are exemplified in Figure 3. Two additional hours of television time (3 h per day compared to 1 h) were associated with a similar pulse rate in participants with low physical activity level (level 1), except for the 68-year-olds having higher pulse rate with 3 h compared to 1 h television time per day. A physical activity level of 4 compared to 1 was associated with lower pulse rate in participants with the same, prolonged television time (3 h), except for the oldest females having a similar pulse rate at level 1 and at level 4.

### Sensitivity analyses

The sensitivity analyses showed no essential differences from the main results.

## Discussion

This study showed that a higher physical activity was associated with a lower pulse rate, while a prolonged television time was associated with a higher pulse rate. Among participants with a prolonged television time, those with a high physical activity had a lower pulse rate compared to those with a low physical activity. Consequently, our results suggest that physical activity could compensate for a prolonged television time regarding pulse rate.

### Higher physical activity is associated with reduced pulse rate

We found that higher physical activity was associated with lower pulse rate in all ages and both sexes. Our results are in line with previous studies demonstrating that increasing physical activity lowers resting pulse rate (3, 7–12, 25). For instance, in an intervention study, two 10-week periods of high- or low-intensity training in 36 middle-aged participants resulted in reduced pulse rates (-5 bpm) (8). These reductions are comparable with the ones observed in the present study when comparing pulse rates of active (level 4) and inactive (level 1) middle-aged females (-2.7 bpm) and males (-4.0 bpm).

### Prolonged television time is associated with higher pulse rate

The present study demonstrated that a prolonged television time was associated with a marginally higher pulse rate. We are not aware of any study examining the effects of television time on pulse rate. Weaker relationships were observed for television time than for physical activity regarding pulse rate, and the reasons behind this are not obvious. Interventional studies will be required to elucidate the effects of prolonged sitting time on pulse rate in more detail.

### High physical activity compensates for prolonged television time

Our results suggest that persons who spend a lot of time watching TV can lower their pulse rate by performing more exercise. In the present study, a higher physical activity level (level 4 instead of level 1) was associated with lower pulse rate in middle-aged females (-2.6 bpm) and males (-3.7 bpm) with prolonged television time (3 h per day). These results correspond well with another study (26) where sedentary subjects were randomized to high-volume training with six sessions per week lasting 60 minutes for a period of eight weeks. Resting pulse rate decreased from 70 ± 7 bpm (mean ± standard deviation) to 60 ± 6 bpm. A meta-analysis showed that 12 or more weeks of high-intensity interval training consisting of short interval sessions of vigorous exercise (30 s to 4 min) decreased resting pulse rate in overweight/obese groups (-0.33 bpm) (7). Taken together, there is evidence that exercise can reduce pulse rate and consequently cardiovascular risk in sedentary and obese individuals.

### Interaction between age and physical activity regarding pulse rate

Another interesting finding is that age interacted with physical activity regarding pulse rate. The physical activity versus pulse rate relationship was more pronounced in the young compared to the elderly. This indicates that it is easier for young people to reduce their pulse rate by increasing physical activity. It has been noted previously that an intervention by exercise can lower the pulse rate in young and middle-aged persons (25, 27), but not in elderly (28, 29).

### Perspectives

Compared to sedentary individuals, subjects with a high physical activity had a 4–5 bpm lower pulse rate. A recent meta-analysis (30) described that for every 10 bpm increase of resting pulse rate, the risk of all-cause and cardiovascular mortality increases with 8% and 9%, respectively. However, it was found that only a pulse rate higher than 90 bpm is associated with a significant increased risk of cardiovascular mortality. The relationships observed in our study are in the interval of 60–75 bpm, being below this threshold. It is therefore unclear how our results are related to the risk of cardiovascular mortality in this population. Nevertheless, as a low pulse rate is related to improved health outcomes, our study supports the idea that an increased amount of exercise could enhance health. The findings of our study can be used to encourage individuals with a sedentary lifestyle to increase the physical activity even if the sitting time remains long.

### Strengths and limitations

The strength of the present study is the large sample size including a wide age-span, which allowed the evaluation of high-order interactions with a good statistical power. To our knowledge, the interplay between physical activity and television time on pulse rate in different ages and sexes has not been studied in this large-scale setting before.

A limitation of this study is its cross-sectional design and the subjective estimation of physical activity and television time. Subjective estimation of physical activity can both over- and underreport physical activity compared to objective measurements (31). Self-reported television time may not accurately reflect total sitting time, but sitting time was not uniformly evaluated in LifeGene and EpiHealth and could therefore not be used in the present study. A more objective and precise way to quantify movement would have been by using accelerometry. Moreover, highly educated participants are overrepresented in the sample, as can be seen in Table I, and almost all participants are Caucasians, and therefore our results cannot be generalized to all other populations.

## Conclusion

Physical activity was inversely associated with pulse rate, while television time (as a proxy for sitting time) appeared to have less impact on pulse rate. High compared to low physical activity was associated with a lower pulse rate in subjects with a prolonged television time. We also conclude that it seems easier to reduce pulse rate by doing more exercise for young than for old people.
